# Chemical Composition and Antioxidant Activities of *Broussonetia papyrifera* Fruits

**DOI:** 10.1371/journal.pone.0032021

**Published:** 2012-02-28

**Authors:** Jie Sun, Shao-fang Liu, Chu-shu Zhang, Li-na Yu, Jie Bi, Feng Zhu, Qing-li Yang

**Affiliations:** Shandong Peanut Research Institute, Qingdao, People's Republic of China; New York State Museum, United States of America

## Abstract

Fruits of *Broussonetia papyrifera* from South China were analyzed for their total chemical composition, and antioxidant activities in ethanol and aqueous extracts. In the fruit of this plant, the crude protein, crude fat and carbohydrates was 7.08%, 3.72% and 64.73% of dry weight, respectively. The crude protein, crude fat and carbohydrates were 15.71%, 20.51% and 36.09% of dry weight, respectively. Fatty acid and amino acid composition of the fruit were analyzed. Unsaturated fatty acid concentration was 70.6% of the total fatty acids. The percentage of the essential amino acids (EAAs) was 40.60% of the total amino acids. Furthermore, *B. papyrifera* fruit are rich in many mineral elements and vitamins. Total phenolic content was assessed using the Folin-Ciocalteau assay, whereas antioxidant activities were assessed by measuring the ability of the two extracts to scavenge DPPH radicals, inhibit peroxidation, and chelate ferric ions. Their reducing power was also assessed. Results indicated that the aqueous extract of *B. papyrifera* was a more potent reducing agent and radical-scavenger than the ethanol extract. GC–MS analysis of the ethanol extract showed the presence of some acid-containing compounds. The changes in total phenolic content and antioxidant capacity in *B. papyrifera* from four different regions grown under normal conditions were assessed. The antioxidant activity of different extracts was positively associated with their total phenolic content. These results suggest that the fruit of *B. papyrifera* could be used in dietary supplement preparations, or as a food additive, for nutritional gain, or to prevent oxidation in food products.

## Introduction

Plants have long been used as a source of traditional medicines to treat various diseases and conditions. Many of these medicinal plants are also excellent sources of phytochemicals, many of which have potent antioxidant activities. *Broussonetia papyriferais* is a deciduous tree or shrub which grows naturally in the Asian and Pacific region in China, Thailand and the USA. The roots, leaves, bark and fruit are all used in traditional Chinese medicines. In China, the leaves have been used in folk medicine against chronic prostatitis, and the fruit has been used to treat impotence and ophthalmic disorders, the efficacy of which has been proved by pharmacological experiments [1]. Various compounds identified in this plant have been shown to inhibit lipid peroxidation [2], have antiplatelet effects [3], and inhibit the activities of the protein tyrosine phosphatase 1b (PTP1B) enzyme and aromatase [1], [4].

At present, there is an increasing interest both in industry and scientific research in useful plants which could be recognized as a nutritious food or dietary supplement as well as an important source of biologically active compounds of medicinal value [5], [6]. Abundant nutrient content of the plants help keep our body functioning well. Dietary antioxidants are important components because they protect against free radicals in the human body, such as reactive oxygen species [7]. In recent years, many studies have focused on the properties and composition of extracts obtained from the roots, bark and leaves of *B. papyriferais*. Rong Min Chen isolated three kinds of PTP1B inhibitors from its roots, and Son et al isolated a new prenylated flavonol, papyriflavonol A, from its root bark [3], [8]. However, there are few studies on the chemical composition and antioxidant activities of this plant's fruit. The fruit of *B.papyrifera* is an important source of folk drugs in the Middle East [1], and further research on it would be of great interest. In view of the limited data on this fruit, we set out to: 1) study its chemical composition; 2) evaluate the antioxidant activities of different fruit extracts using several antioxidant assays; and 3) identify candidate constituents responsible for this activity.

## Materials and Methods

### Ethics statement

In this study, no specific permits were required for the described field studies. The study is not privately-owned or protected in any way. The field studies did not involve endangered or protected species.

### Materials

The principle reagents used, and their sources were as follows. 2, 20-diphenyl-1-picrylhydrazyl (DPPH), ferrozine, tocopherol, p-coumaric acid, and linoleic acid were purchased from Sigma-Aldrich (St Louis, MO, USA). Folin-Ciocalteau was sourced from AppliChem (GER). All other reagents were either of analytical grade, or of the highest quality available.

### Plant materials

The ripe fruit of *B. papyrifera*, dark red in colour, were collected from the Zhong shan Arboretum, Qingdao Shandong Province, PR China, in September 2009. The fruits (200 g from 12 different trees) were dry heated in a hot air oven at 50°C for 24 h and the beans were allowed to cool at room temperature. Sample fruit were shelled and broken, before freeze-drying to constant weight, and then powdered to pass through a 40 mesh sieve. The *B. papyrifera* fruits from different regions (Hena Province, Hubei Province, Guangxi Province) were bought from traditional Chinese medicine pharmacy in Qingdao.

### Proximate and mineral analyses

Proximate analysis, including moisture, crude fat, fiber, ash, and crude protein contents, was performed in three biological replicates, according to Association of Official Analytical Chemists AOAC (1995) [9] procedures. For mineral analyses, a Hitachi Z-8000 atomic absorption spectrometer procedure, described by AOAC (1995), was used. Phosphorus content was measured by phosphorus molybdenum blue spectrophotometry. Pb and Cd levels in samples were determined by a HGA graphite furnace, using argon as the inert gas. Other measurements were carried out in an air/acetylene flame. V_B1_, V_B2_, V_B5_ and V_B6_ contents were measured by spectrophotometer. V_C_, ß- carotene, V_D_, V_E_ and V_A_ levels in samples were measured by high performance liquid chromatography [10].

### Fatty acid analysis

Samples (150 mg) were mixed with heptadecanoic acid methyl ester (internal standard) and extracted with chloroform/methanol (2∶1) at 60°C for 1 h. The final extract was concentrated to 5 mL. Fatty acids in the extract were simultaneously hydrolyzed and derivatized as methyl esters with 1 mL of NaOH/methanol at 90°C for 10 min, and then a complete derivation was assured with 1 mL of BF_3_ at 90°C for 10 min. The methyl esters were purified with 1 mL of hexane and 1 mL of water. Individual samples were passed through an anhydrous Na_2_SO_4_ column, and then evaporated to dryness under a stream of nitrogen and re-dissolved in 100 µL of isooctane. The derivatized fatty acids were separated in a HP5890 Series II gas chromatograph equipped with a MS detector 5972 and a cross-linked column with a stationary phase of 5% phenyl methyl silicone [11].

### Amino acid analysis

A modification of the AOAC (1995) method was used for amino acid analysis. Dry samples were hydrolyzed with 25 mL 6N HCl at 110°C for 24 h. Amino acid analysis was carried out by ion-exchange chromatography in an automatic amino acid analyzer (Hitachi L-8800).

### Preparation of plant extracts

Fruit powders (50 g) were extracted by using a Soxhlet extractor for 1 h with 50 ml of extractant, including petroleum ether under reflux conditions. The drying residues (10 g) were then extracted with ethyl alcohol, at room temperature for 24 h with a mass to volume ratio of 1∶20 (g/mL) and filtered through Whatman No. 4 filter paper. The extracts were evaporated to dryness on a rotary evaporator at 40°C. After ethyl alcohol extracted the residues were then treated with water under the same conditions. The ethyl alcohol and aqueous extracts were subsequently re-dissolved in the same solvent at a concentration of 5 mg/ml. Extracts were kept at 4°C until the bioassay analyses.

### Total phenolic analysis

The Folin-Ciocalteau method was used for total phenolic analysis [12], [13]. Ten milliliters of 1∶10 Folin-Ciocalteau reagent was added to 100 µL of sample (5 mg/ml) or standard (5 mg/ml). The mixture was mixed and incubated for 5 min before the addition of 2.5 mL of 2%Na_2_CO_3_. The resulting solution was incubated for a further 2 h before absorbance readings were taken at 760 nm. Gallic acid was used for the calibration curve and a range of concentrations from 100–1000 mg/L were prepared and analyzed as above. Results were expressed as mg gallic acid equivalents (GAE)/g dried plant material. All experiments were done in triplicate.

### Antioxidant activity assays

#### Determination of 2, 20-diphenyl-1-picrylhydrazyl (DPPH) radical scavenging ability

The scavenging activity of the ethanol and aqueous extracts on DPPH radicals was measured according to the method of Tuberoso with some modifications [14], [15]. 250 µL of extract, at different concentrations (0.5–5 mg/ml), were dissolved in 2.5 ml of DPPH, 0.1 mmol/l in methanol. Ethanol or water was used instead of the extract samples as a control. Spectrophotometric readings were carried out with a Cary 50 spectrophotometer at 517 nm, using a 10 mm quartz cuvette. Standards were prepared in a similar manner with ascorbic acid, tocopherol and 2, 6-Di-tert-butyl-4-methylphenol (BHT) at different concentrations, for comparison. All experiments were done in triplicate.

The scavenging activity (%) (SA) on DPPH radicals was calculated using the formula:
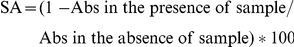



#### Chelation of metal ions (Fe^2+^)

The ability of the ethanol and aqueous extracts to chelate Fe^2+^ ions was evaluated using the method of Dinis, Madeira and Almeida (1994) with some modification [16]. 300 µL of sample, at various assay concentrations (0.5–5 mg/ml) was made up to 3.7 ml with deionized water. A solution of 2 mM ferrous chloride (0.1 ml) was added, and after 3 min the reaction was inhibited by the addition of 5 mM ferrozine (0.2 ml). The mixture was shaken vigorously and left at room temperature for 10 min. Absorbance of the resulting solution was measured at 562 nm. A blank without the sample was prepared in a similar manner. Standards with ascorbic acid, tocopherol and 2, 6-Di-tert-butyl-4-methylphenol (BHT) at various concentrations were also made in a similar manner for comparison. All experiments were done in triplicate.

The chelating capacity was calculated as follows:
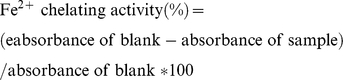



#### Reducing power

The reducing power was measured following Oyaizu (1986) with some modification [17]. 250 µL of extract with different concentrations (0.5–5 mg/ml) were mixed with 1.75 ml of 0.2 M phosphate buffer (pH 6.6) and 1 ml of 1% potassium ferricyanide. The mixture was incubated at 50°C for 20 min followed by the addition of 1 ml 10% TCA. An aliquot (1 ml) from the incubation mixture was mixed in a test tube with 1 ml of distilled water and 0.2 ml of 0.1% ferric chloride. After 10 min the absorbance of the resulting solution was measured at 700 nm. An increased absorbance of the reaction mixture indicates increased reducing power [18]. A standard with ascorbic acid, tocopherol and 2, 6-Di-tert-butyl-4-methylphenol at various concentrations was also made in a similar manner for comparison. All experiments were done in triplicate.

#### Measurement of the lipid peroxidation inhibition activity in a linoleic acid emulsion system

The lipid peroxidation inhibition activity of the ethanol and aqueous extracts were measured in a linoleic acid emulsion system according to the methods of Qian, Jung, Byun, and Kim (2008) with some modification [19]. Briefly, 2.0 ml of extracts (0.5–5 mg/ml) was mixed with 2 ml of 2.5% linoleic acid dissolved in 95% ethanol. Then, 4 ml of 50 mM sodium phosphate buffer (pH 7.0) and 2 ml of distilled water were added. The mixture was incubated in a 20 ml centrifuge tube at 40±1°C in a dark room, and the degree of oxidation was evaluated by measuring the FeSCN values described below. The reaction solution (0.1 ml), incubated in the linoleic acid model system, was mixed at different intervals during the incubation period with 9.7 ml of 75% ethanol, 0.1 ml of 30% NH_4_SCN, and 0.1 ml of 20 mM FeCl_2_ solution in 3.5% HCl. After 3 min, the SCN value was measured by reading the absorbance at 500 nm. An equivalent volume of distilled water was used as a blank [20]. Ascorbic acid, tocopherol and 2,6-Di-tert-butyl-4-methylphenol (BHT) at 0.5–5 mg/ml was used as a control. All experiments were done in triplicate.
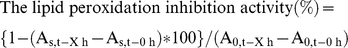
Where: A_s,t-X h_ and A_s,t-0 h_ are the absorbances of the sample at X h and 0 h, respectively; and A_0,t-X h_ and A_0,t-0 h_ are the absorbances of the blank at X h and 0 h, respectively. Plots were made of scavenging activity against the concentration of the sample at 96 h and 168 h.

#### Antioxidant activity analysis of ethyl alcohol and aqueous extracts of *B. papyrifera* fruit from different regions

The antioxidant activity assays were performed on the extracts of *B. papyrifera* fruit from different regions (Hebei Province, Hubei Province, Jiangxi Province and Shandong Province). The ethyl alcohol and aqueous extracts of *B. papyrifera* fruit from different regions were obtained according to the methods described in 2.6. The scavenging activity, Fe^2+^ chelating activity, reducing power and lipid peroxidation inhibition activity of ethyl alcohol and aqueous extracts from different regions were detected by the methods described in 2.8.1, 2.8.2, 2.8.3. All experiments were done in triplicate.

### Measurements by high-performance liquid chromatography (HPLC)

0.01 g of dried ethanol and aqueous extracts were mixed with 2 mL of acetonitrile and 0.2 mL of 0.1 Nhydrochloric acid and stirred for 2 h at room temperature. The suspension was centrifuged at 10000 rcf for 1 min. The analyses were performed using a Waters Sun Fire C18 column (4.6×250 mm) and a 2998 diode matrix detector in a Waters e2695 HPLC system. The mobile phase was methanol (solvent A) and 5mlphosphate (solvent B). The gradient was 0–25 min, 20%–80% A, 80%–40% B. The mobile phase was 0.1% phosphate/methanol at a flow rate of 0.8 mL/min, and UV detection was at 273 nm. Phenolic acid standards, such as 7-hydroxycoumarin, ferulic acid, resveratrol, rutin, cinnamylate, meletin, protocatechuate acid, catechuic acid, epicatechin, protocatechuic acid and p-hydroxy phenyl ethyl ketone, were used for the identification of phenolic acids present in the *B. papyrifera* extracts. Sample compounds were identified on the basis of the retention times of standard materials and were quantified by comparing their peak areas with those of standard curves [21].

### Statistical analyses

The SPSS 13.0 software (SPSS Inc., Chicago, IL, USA) was used to calculate the means and standard deviations in any experiments involving duplicate or triplicate analyses of any sample or condition. The statistical significance of any observed differences was evaluated by oneway analysis of variance (One-way ANOVA), using the Bonferroni Multiple Comparisons Test.

## Results and Discussion

### Proximate and mineral analyses

The proximate composition of fruits of *B. papyrifera* is shown in Table 1, and the following data are expressed as average percentage dry weight (DW). The moisture content of the fruit was 3.75%. Carbohydrate (36.09%) formed the single largest component in comparison to other constituents detected. Crude fat content (20.51±0.21) was much higher compared to other fruit such as *Citrullus lanatus var* (10.9%) [22] and white mahlab *Prunus mahaleb* (13.15%) (P<0.05) [23]. which indicated very clearly that the *B. papyrifera* formed a potential source of oils, and fats. However, it would be seen that, the fat content was lower than most Chinese conventional oil fruits (sunflower 34.4%, sesame 59.6% and groundnut 40.1%) (P<0.05) [22]. Crude protein content was 15.71%, which was less than the range of most conventional oil seeds, such as sesame, bean, and sunflower which contain about 18.3–25.3% protein [24].

**Table 1:** Moisture content and proximate composition of *B. papyrifera* fruits (g/100 g DW).

component	moisture	ash	crude fat	crude protein	carbohydrates	crude fiber
fruits	3.75±0.17	9.74±0.13	20.51±0.21	15.71±0.16	36.09±0.32	14.20±0.23

The levels of trace elements in the fruit are shown in Table 2. *B. papyrifera* fruit were found to be a good source of minerals. Calcium content was 17338.9±15.12 mg/kgDW, whereas the magnesium content was 3284.6±12.1 mg/kgDW. Calcium is important for bone growth, and muscle and neurological function, whereas magnesium is linked with the prevention of ischemic heart disease. The *B. papyrifera* fruit had a high level of potassium (9098.7±14.18 mg/kgDW). Potassium is known to play an important role in regulating the permeation pressure of cells and takes part in the metabolism of sugar and protein in human cells in vitro. These fruit could theoretically provide other nutritionally useful minerals including copper, iron, magnesium and zinc (Table 2). The levels of iron and copper correspond with the recommended dietary allowances of NRC/NAS.

**Table 2:** Mineral composition of *B. papyrifera* fruits (mg/kg DW).

minerals	fruits
Ca mg/kg	17338.9±15.12
Cu mg/kg	19.75±0.43
Fe mg/kg	414.8±8.23
Mg mg/kg	3284.6±12.1
Zn mg/kg	29.8±1.01
Se µg/kg	<0.08±0.00
Cr mg/kg	3.48±0.11
K mg/kg	9098.7±14.18
Mn µg/kg	44.56±0.76
Cd mg/kg	0.009±0.00
Pb mg/kg	0.52±0.03
P mg/kg	4644.2±12.98
Hg µg/kg	4.91±0.79

Table 3 shows the vitamin composition of the fruit analyzed. Of the nine vitamins analyzed, seven were found at various levels. V_A_ and V_D_ could not be detected in this species. V_E_ is a free radical scavenger, which can prevent damage to the body by free radicals, and was present in the fruit at a concentration of 7.29 mg/100 g.

**Table 3:** Vitamin composition of *B. papyrifera* fruits (mg/100 g).

vitamin	ß- carotene	V_E_	V_D_	V_A_	V_B1_	V_B2_	V_B5_	V_B6_	V_C_
fruits	0.09±0.00	7.29±0.054	-	-	0.32±0.023	0.12±0.01	1.85±0.017	1.21±0.09	0.01±0.00

Table 4 shows the fatty acid composition of the fruit. C18:2, C18:0, and C16:0 were the main fatty acid constituents. C18:2 is an essential fatty acid, which can reduce cholesterol levels in the blood. C18:2 constituted 52.5833% of the total fatty acids in the fruit. The C18:2 content was much higher than that of rapeseed, peanut and sesame [22]. These results suggest that the fruit of *B. papyrifera* could be a good source of C18:2 in the human diet. Other fatty acids, for example, C10:0, C12:0, C17:0 and C23:0 were found only in very small amounts.

**Table 4:** Fatty acid composition (%) of *B. papyrifera* fruits.

fatty acids	fruits
C9:0	0.2316±0.0023
C10:0	0.0182±0.0009
C12:0	0.0766±0.0013
C 14:0	0.7742±0.0033
C 15:0	0.2375±0.0021
C 16:1 (cis - 9)	0.6219±0.0043
C 16:0	16.3625±0.0098
C 17:0	0.2374±0.0003
C 18:2 (cis,cis - 9,12) (omega 6)	52.5833±0.0148
C 18:1(cis - 9) (omega 9)	16.9175±0.0072
C 18:0	3.7695±0.0042
C 20:1 (cis - 11) (omega 9)	0.4953±0.0024
C 20:0	2.0615±0.0044
C 21:0	0.3348±0.0012
C 22:0	4.0033±0.0035
C 23:0	0.2011±0.0003
C 24:0	1.0757±0.0053

Sixteen known amino acids were found in the fruit proteins of *B. papyrifera,* as shown in Table 5. The essential amino acids (EAAs) comprised 40.59% of the total, and the ratio of EAAs to non-EAAs in the fruit was 0.67, only slightly higher than the reference values of 40% and 0.6 recommended by FAO/WHO [25].

**Table 5:** Amino acid composition of *B. papyrifer*a fruits (mg/100 mg of Protein).

amino acid	fruits
aspartate	1.61±0.23
threonine	1.67±0.43
serine	0.74±0.17
glutamate	2.46±0.21
glycine	1.07±0.15
alanine	0.61±0.12
cysteine	-----
valine	0.81±0.24
methionine	0.05±0.02
isoleucine	0.65±0.18
leucine	1.05±0.27
tyrosine	0.48±0.16
phenylalanine	0.76±0.16
lysine	0.71±0.17
histidine	0.34±0.12
arginine	0.71±0.11
proline	0.32±0.14

### Total phenolic analysis

The total phenolic content of the two types of fruit extract were determined following a modified Folin-Ciocalteu reagent method and the results were expressed as gallic acid (GA) equivalents. The aqueous extract of the dried fruit had the highest phenolic content (5.79±0.12 mg GAE/g), followed by the ethanol extract (3.79±0.03 mg GAE/g).

### DPPH radical scavenging activity

**Figure 1:**
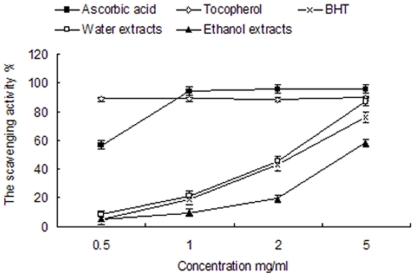
DPPH radical scavenging capacity of ethanol and aqueous extracts of *B. papyrifera*. The activity of different amounts (0.5–5 mg/ml) of ascorbic acid, tocopherol, BHT, ethanol extracts and aqueous extracts is expressed as per cent scavenging and the values represents the means of three independent experiments.

The DPPH radical inhibition assay is a widely used and comparably easy method to evaluate antioxidant activity, because DPPH is a stable free radical, which produces a violet solution in ethanol. It is reduced and decolorized in the presence of an antioxidant molecule [26]. Using this method, the radical scavenging activities of the fruit extracts of *B. papyrifera* were estimated by comparing the percentage inhibition of formation of DPPH radicals by the extracts compared to those of standards. Fig. 1 shows a steady increase in the percentage inhibition of the absorbance of the DPPH radicals by the extracts up to a concentration of 5 mg dried extract/mL. This pattern of DPPH inhibition is commonly observed with plant extracts [27]. The ethanol and aqueous extracts of *B. papyrifera* were able to inhibit the formation of DPPH radicals with a percentage inhibition of 87.17±0.18, and 58.11±0.11% at the highest concentration, respectively (Fig. 1). When compared with standards, the DPPH radical scavenging capacity of the aqueous extract was weaker than ascorbic acid and tocopherol, and higher than BHT at the same concentration. DPPH radical scavenging capacity of the aqueous extract was weaker than all of standards we tested, at the same concentration.

### Chelation of metal ions (Fe^2+^)

**Figure 2:**
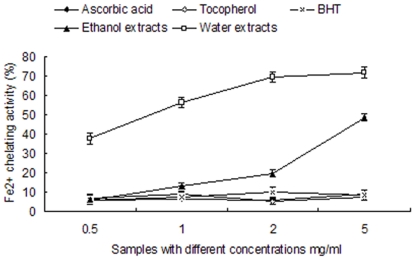
Fe^2+^-chelating activity of ethanol and aqueous extracts of *B. papyrifera*. Determination of Fe^2+^-chelating activity of ascorbic acid, tocopherol, BHT, and different concentrations (0.5–5 mg/ml) of ethanol extracts and aqueous extracts. The activities are expressed as % chelating and monitored in three biological replicates.

In the present study, the Fe^2+^-chelating activities of the different extracts were compared with that of Ascorbic acid, tocopherol and 2,6-Di-tert-butyl-4-methylphenol (BHT) (Fig. 2). The Fe^2+^-chelating activities of the ethanol and aqueous extracts of *B. papyrifera* fruit were excellent and increased steadily with increasing concentration. With an Fe^2+^-chelating activity of approximately 77.51%, the aqueous extract (5 mg/ml) showed significantly higher Fe^2+^-chelating activity than the three standards, at the same concentration (P<0.01). The ethanol extract (5 mg/ml), showed a chelation capacity of 48.26%, and also showed a higher Fe^2+^-chelating activity than the three standards (P<0.05). This result clearly shows that the lower molecular weight fractions contained some special components that can chelate metal ions such as Fe^2+^.

### Reducing power

**Figure 3:**
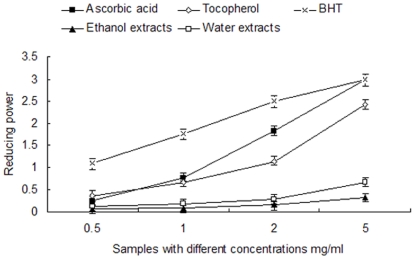
Reducing power capacity of ethanol and aqueous extracts of *B. papyrifera*. Ascorbic acid, tocopherol, BHT, ethanol and aqueous extracts at various concentrations (0.5–5 mg/ml) were used to detect the reducing power capacity. The reducing power capacities of different samples are monitored in three biological replicates.

The reducing power of the ethanol and aqueous extracts are shown in Fig. 3. In this assay, the yellow color of the test solution changes to various shades of green and blue, depending on the reducing power of each compound. The presence of reducers (i.e., antioxidants) causes the reduction of the Fe^3+^ ferricyanide complex to the ferrous form. Therefore, measuring the formation of Perl's Prussian blue at 700 nm can monitor the Fe^2+^ concentration [21]. The ethanol and aqueous extracts showed lower reducing power when compared to the three standards. The aqueous extract showed higher reducing power than the ethanol extract at the same concentration. These findings indicate that the two kind of extracts contained minute quantities of compounds with high reducing power.

### Measurement of the lipid peroxidation inhibition activity in a linoleic acid emulsion system

**Figure 4:**
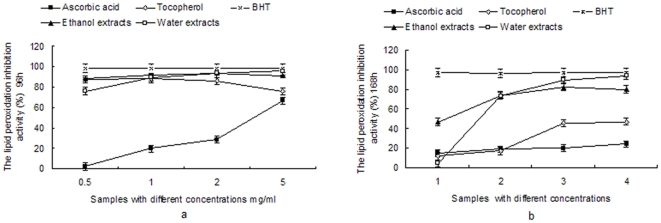
The lipid peroxidation inhibitory activity of ethanol and water extracts of *B. papyrifera*. a, The lipid peroxidation inhibitory activity of ascorbic acid, tocopherol, BHT, ethanol and aqueous extracts at various concentrations(0.5–5 mg/ml) in 96 h. b, The lipid peroxidation inhibitory activity of ascorbic acid, tocopherol, BHT, ethanol and aqueous extracts at various concentrations(0.5–5 mg/ml) in 168 h.

The degree of lipid peroxidation inhibition of the ethanol and aqueous extracts at different times (96 h and 168 h) are shown in Fig. 4. Lipid peroxidation is thought to proceed via a radical-mediated abstraction of hydrogen atoms from methylene carbons in polyunsaturated fatty acids [28]. The amount of lipid peroxidation inhibition of the ethanol and aqueous extracts in the linoleic acid system was similar with that of tocopherol, and higher than ascorbic acid after 96 h. Nevertheless, the degree of lipid peroxidation inhibition of the two extracts was much higher than that of tocopherol and ascorbic acid after 168 h (P<0.05). That is to say that both the ethanol and aqueous extracts had a stronger lipid peroxidation inhibitory effect than tocopherol and ascorbic acid at the same molecular concentration, when the experiments were allowed to run for an extended period of time.

### Antioxidant activity analysis of ethyl alcohol and aqueous extracts of *B. papyrifera* fruit from different regions

**Figure 5:**
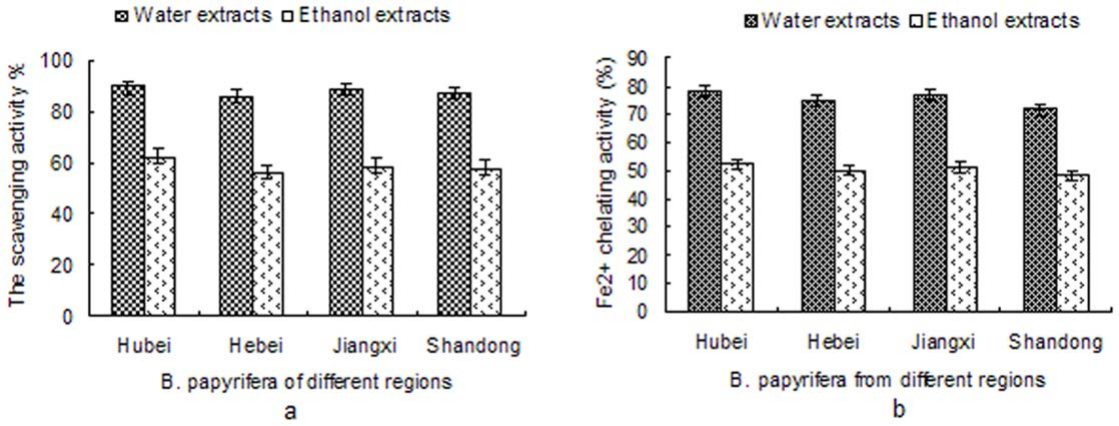
The scavenging activity and Fe^2+^ chelating activity of ethyl alcohol and aqueous extracts of *B. papyrifera* from different regions. a, DPPH radical scavenging capacity of ethanol and aqueous extracts (5 mg/ml) from different region (Hubei,Hebei,Jiangxi and Shandong Province). b, Fe^2+^ chelating activity of ethanol and aqueous extracts (5 mg/ml) from different region (Hubei,Hebei,Jiangxi and Shandong Province).

**Figure 6:**
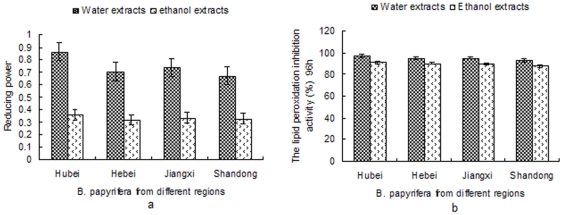
The reducing power and lipid peroxidation inhibition activity of ethyl alcohol and aqueous extracts from different regions. a, The reducing power of ethanol and aqueous extracts (5 mg/ml) from different region (Hubei,Hebei,Jiangxi and Shandong Province). b, Lipid peroxidation inhibition activity of ethanol and aqueous extracts (5 mg/ml) from different region (Hubei,Hebei,Jiangxi and Shandong Province).

The antioxidant capacity of ethanol and aqueous extracts of *B. papyrifera* fruit from different regions (Hubei,Hebei,Jiangxi and Shandong Province) was evaluated in order to establish how environmental influences could affect it, by using four different methods: DPPH, Fe^2+^ chelating activity, reducing power and lipid peroxidation inhibition activity tests (Fig. 5, 6). The total phenolic content of the all types of fruit extract were determined (Table 6). The cavenging activity and Fe^2+^ chelating activity of the eight samples were given in fig. 5. The four aqueous extracts or ethanol showed no significant differences (P<0.05) in the two analyzed tests. The ethanol and aqueous extracts from Hubei province showed higher levels of scavenging activity and Fe^2+^ chelating activity, while the extracts from Shangdong province showed lower levels of antioxidant activity. The reducing power and lipid peroxidation inhibition activity of ethyl alcohol and aqueous extracts from different regions were showed in fig. 6. The results showed that the reducing power and lipid peroxidation inhibition activity of Hubei and Jianhxi province were higher than those in the extracts derived from fruit originating from the other provinces. However, there are no significant differences (p<0.05) in all analyzed ethanol and aqueous extracts respectively. The results from antioxidant activity analysis of extracts from four different regions revealed that the different growing environment had certain effect on antioxidant activity, but the impact is not big. And the antioxidant activity of the extracts had positive correlation with the total phenolic content of the samples. The total phenolic content of different extracts showed a strong correlation their reducing power (R2 = 0.932, P<0.05) and lipid peroxidation inhibition activity (R2 = 0.96, P<0.01).

**Table 6:** Total phenolic content of *B. papyrifera* fruits extracts from different regions (mg GAE/g).

regions	Hubei		Hebei		Jiangxi		Shandong	
extracts	water	ethanol	water	ethanol	water	ethanol	water	ethanol
total phenolic	6.13±0.17	4.18±0.11	5.94±0.13	4.02±0.51	5.91±0.21	3.841±0.32	5.79±0.1	3.79±0.03

### High-performance liquid chromatography (HPLC) measurements

The antioxidant activities observed were associated with the total phenolic content, but the contribution of each phenolic compound to this activity should be evaluated. HPLC analysis was therefore performed on the two extracts from *B. papyrifera* fruit. The HPLC analysis is key to understanding the relationship between the compounds discovered and their activities.

In the present study, twelve components were analyzed as standards in the HPLC analysis, and four phenolic components were found in the ethanol and aqueous extracts. The four components were identified as 7-hydroxycoumarin, protocatechuate acid, protocatechuic acid and epicatechin. In addition, ferulic acid was found in the ethanol extract only. These results suggested that the ethanol and aqueous extracts had a similar phenolic compound composition, but at different concentrations. This might explain the differences in the antioxidant activities of the two extracts. Protocatechuate acid and protocatechuic acid have been found in many plants. These compounds possess strong antioxidative, antibacterial, and antimutagenic properties. 7-hydroxycoumarin has been found to have antioxidative properties in many plants such as *Fraxinus ornus* and *Eysenhardtia subcoriacea* [29], [30]. Hu confirmed that the 7-hydroxycoumarin group could contribute to the antioxidative activity of coumarin *via* an electron transfer mechanism [31]. Also, ferulic acid is found in many Chinese medicinal herbs and is an effective antioxidant and vasodilatory agent.

### Conclusion

In conclusion, our data suggest that *B. papyrifera* fruit are an excellent source of fatty acids, essential in the human diet. They also contain considerable amounts of carbohydrates, proteins and a number of minerals including calcium, magnesium, and copper. These assays also show that these fruit contain very useful phytochemicals such as7-hydroxycoumarin, protocatechuate acid, ferulic acid, protocatechuic acid and epicatechin and have substantial antioxidant activity. Phenolic compounds and flavonoids appear to be the main components responsible for the antioxidant activity of the fruit extracts. The combination of bioactive compounds and rich nutritional composition of these fruit make them valuable as functional food or as nutritional supplements.
